# Palmer Arm and Leg—Manufactured under the Direction of the Inventor and Patentee

**Published:** 1866-04

**Authors:** B. Frank Palmer

**Affiliations:** President of the American Art. Limb Company


*Palmer Arm and Leg—Manufactured under the direction of the Inventor and patentee*, B. Frank Palmer, L. L. D., President of the American Art. Limb Company.

We are in receipt of a pamphlet of 48 pages from the above company, most of which is occupied with the testimony of surgeons, generals, soldiers and private citizens, in favor of the productions of this manufactory. Soldiers of the United States army are furnished with artificial limbs from this and other manufactories, at government expense.

Soldiers of the Southern army, being left out of this programme, must needs go on crutches, if unable to buy, until the States adopt some plan by which they can be furnished. The Georgia Legislature, we find, has appropriated money for this purpose, and we hope arrangements will be made soon, that will make artificial limbs available to our maimed soldiers. We have not yet had an opportunity of inspecting the Palmer limb, but from the array of testimonials in the publication before us, it is, undoubtedly, all that could be desired.

*Valuable Medical Works.*—We are in receipt of Lindsay & Blakiston's Catalogue of Medical and scientific publications, Philadelphia. In looking through the catalogue, we find the "Epitome of Braithwait's Retrospect—the cream of forty volumes of the Retrospect up to 1860"—in two large octavo volumes of 900 pages each, by Walter S. Wells, M. D.—Price $10.00.

Before the war it was our good fortue to become possessed of the "Epitome" in six volumes of about 300 pages each, and found it almost a library of medicine and surgery in itself. We cannot too highly recommend it to the shelves of every physician's office.

We find in the catalogue an extensive list of standard medical works.

